# Lentivirus-Induced Dendritic Cells (iDC) for Immune-Regenerative Therapies in Cancer and Stem Cell Transplantation

**DOI:** 10.3390/biomedicines2030229

**Published:** 2014-08-21

**Authors:** Renata Stripecke

**Affiliations:** Regenerative Immune Therapies Applied, Excellence Cluster Rebirth, Department of Hematology, Hemostasis, Oncology and Stem Cell Transplantation, Hannover Medical School, OE6862, Carl-Neuberg-Strasse 1, 30625 Hannover, Germany; E-Mail: stripecke.renata@mh-hannover.de; Tel.: +49-511-532-6999; Fax: +49-511-532-6975

**Keywords:** lentiviral vectors, dendritic cells, cancer, stem cell transplantation

## Abstract

Conventional dendritic cells (cDC) are *ex vivo* differentiated professional antigen presenting cells capable of potently stimulating naïve T cells and with vast potential for immunotherapeutic applications. The manufacture of clinical-grade cDC is relatively complex and requires several days for completion. Clinical trials showed poor trafficking of cDC from subcutaneous injection sites to lymph nodes (LN), where DC can optimally stimulate naïve lymphocytes for long-lasting memory responses. We demonstrated in mouse and human systems that a single overnight *ex vivo* lentiviral (LV) gene transfer into DC precursors for production of combination of cytokines and antigens was capable to induce autonomous self-differentiation of antigen-loaded DC *in vitro* and *in vivo*. These highly viable induced DC (iDC) effectively migrated from the injected skin to LN, where they effectively activated *de novo* antigen-specific effector memory T cells. Two iDC modalities were validated in relevant animal models and are now in clinical development: Self-differentiated Myeloid-derived Antigen-presenting-cells Reactive against Tumors co-expressing GM-CSF/IL-4/TRP2 for melanoma immunotherapy in the autologous setting (SmartDCtrp2), and Self-differentiated Myeloid-derived Lentivirus-induced against human cytomegalovirus as an allogeneic matched adoptive cell after stem cell transplantation (SmyleDCpp65). The lentiviral vector design and packaging methodology has “evolved” continuously in order to simplify and optimize function and biosafety of *in vitro* and *in vivo* genetic reprogramming of iDC. Here, we address the challenges seeking for new creations of genetically programmed iDC and integrase-defective LV vaccines for immune regeneration.

## 1. Introduction

Numerous clinical trials have been carried out to assess the ability of immunotherapy using conventional dendritic cells (cDC) differentiated *ex vivo* in the presence of different combination of recombinant cytokines. These trials showed to be challenging as only modest objective anti-tumor responses were observed and most approaches failed to move cDC beyond Phase III testing as they were not shown superior to chemotherapy [1,2]. The only FDA-approved cDC-like product is sipuleucel-T, consisting of leukocytes activated with a fusion protein (GM-CSF and the prostatic acid phosphatase) [3]. Noteworthy, in a few cases where the *in vivo* viability and bio-distribution of therapeutic cDC were monitored after administration, low migration (<4%) to lymph nodes was observed and most DC remained at the injection site, lost viability, and were cleared by infiltrating macrophages within 48 h [4]. The low viability and migration capability of cDC may negatively impact antigen (Ag) loading and persistence of the Ag presentation for therapeutic effects. To the current date, treatment of patients with *ex vivo* generated cDC loaded with cell lysates, proteins and peptides is performed mostly within Phase I and II clinical trials. Progression to larger clinical trials is compromised by the high costs of manufacturing, availability of clinical grade reagents (cytokines, toll-like receptor agonists, RNA and antigens), poor consistency and low viability [2,5]. During the last decade, several groups have also explored the transfection/electroporation of DC with messenger RNAs obtained from tumors or expressing stimulatory molecules [6,7]. Multiple RNA transfection of cDCs, however, faces an unpredictability of the stability of transgene expression in DC (h to a few days) because the RNA can be rapidly degraded and RNA pools may result in diminished presentation of individual epitopes [8]. In animal models, RNA transfection of cDC was shown in to be less effective than transduction of cDC with lentiviral vectors (LV) for eliciting therapeutic effects [9]. In face of the general difficulties in clinical development of large amounts in short time of genetically enhanced viable cDC capable to effectively migrate to lymph nodes for orchestrating *de novo* adaptive immune responses, we have explored LV as a tool to reprogram the “next generation” of DC [10]. LV are able to infect DC precursor subsets and cDC with high efficiency in the absence of cytotoxic or unwanted immunologic effects, and their potential use as vectors for gene modification of DC or as direct vaccines has been actively explored [11].

## 2. Lentiviral Vectors (LV) for Robust Genetic Modification of Hematopoietic Cells

Lentiviruses belong to the family of retroviridae that have a diploid, positive-strand RNA genome which is reverse transcribed and permanently integrated in the genome of the host cells. Conversion of these deadly pathogens into effective tools of gene transfer in gene therapy studies were originated from pioneering studies by Naldini *et al*. [12] over a decade ago, which described the capability of engineered replication-deficient lentiviral vectors derived from the Human Immunodeficienty Virus (HIV) in permanently infecting non-dividing cells such as macrophages and neurons [12]. Lentiviral vectors have very favorable characteristics for gene therapy and vaccine development since they infect non-replicating cells delivering up to 7 kb of transgenic cargo, but causing only minor bystander immunological effects. LV have been recently shown to be safe in several gene therapy clinical trials for gene replacement in hematopoieitic stem cells for diseases such as adrenoleukodistrophy [13], Wiskott Aldrich syndrome [14] and metachromatic leukodystrophy [15]. LV expressing a chimeric antigen receptor targeted against CD19 (CAR19) and used for T cell modification showed high efficacy against B cell lymphoma and leukemia [16,17]. Still in stages of pre-clinical development and testing in humanized mouse models for future clinical development, LV expressing a chimeric antigen receptor targeted against adhesive receptor CD44V6 is currently being explored for immunotherapy against acute myeloid leukemia and multiple myeloma [18]. In this setting, in order to avoid graft *versus* host disease and monocytopenias, a co-expressed suicide gene included in the vector enabled pharmacologic ablation of CD44v6-targeted T cells [18]. LV-mediated modification of CD4^+^ T cells has also been experimentally explored in order to induce tolerance, e.g., by constitutive expression of interleukin (IL)-10 [19] or forkhead box P3 (FOXP3) [20]. Therefore, LV are considered a state-of-the-art viral vector platform for robust, consistent and safe genetic modification of hematopoieitic cells [21]. LV have been long considered for the development of vaccines and the further development and validation of bio-safety of lentiviral vectors for immunotherapy of cancer and chronic infections is a topic of broad interest [11].

## 3. Combinations of Transgenes in LV for Reprogramming Precursors into Antigen-Loaded Dendritic Cells (DC)

Granulocyte macrophage colony stimulating factor (GM-CSF), interleukin (IL-4) and Interferon α (IFN-α) are cytokines that have been extensively characterized for *in vitro* differentiation of peripheral blood (PB) CD14^+^ monocytes into cDC. Monocytes require bio-active GM-CSF to yield viable DCs and GM-CSF is considered a critical factor for DC development under both steady-state and inflammatory conditions. GM-CSF works through activation of several signaling modules including JAK/STAT, MAPK, PI3K, and canonical NF-κB influencing the differentiation and survival of DC subset precursors [22]. IL-4 and IFN-α function upon GM-CSF-stimulated monocytes to further acquire the typical activated and terminally differentiated DC immunophenotype (e.g., high expression of HLA-DR and CD86/B7-2). In the lack of bio-functional IL-4 or IFN-α, monocytes incubated only in the presence of GM-CSF *in vitro* differentiate into macrophages [23]. DC obtained with recombinant GM-CSF and IL-4 are not stable mature dendritic cells, and if the cytokines are removed from the medium, the characteristic morphology and non-adherence of DC is lost [24]. Therefore, after a 6–7 day culture with GM-CSF and IL-4, DC require treatment with additional factors to acquire the putative CD83^+^ “mature state”, where they are highly potent at stimulating naïve T cells. On the other hand, several other superior attributes have been given to treatment of DC precursors with GM-CSF and IFN-α such as a higher maturation profile, observed migration of the resulting DC in response to Β-chemokines and capacity to induce potent primary antibody responses [25]. Recombinant GM-CSF, IL-4 and IFN-α were approved for clinical use and extensive pharmacological and safety information is available for humans. Gene therapy trials conducted with cells or virus modified for transient expression of GM-CSF, IL-4 and IFN-α showed no toxic effects. Therefore, we evaluated the LV-mediated transgenic expression of these cytokines to induce monocytes to self-differentiate into DCs.

**Figure 1:**
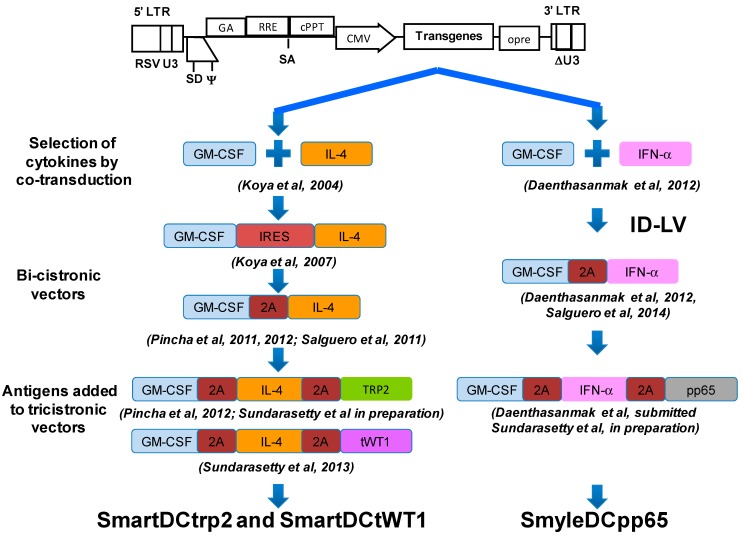
Lentiviral vector designs that enabled the induction of monocytes into long-lived and potent induced dendritic cells (iDC). The backbones contain chimeric 5' long terminal repeats (LTR), the packaging signal (Ψ), the truncated and out-of-frame gag gene (GA), the Rev responsive element (RRE), the central polypurine and termination sequence (cPPT), the cytomegalovirus (CMV) major immediate-promoter, the mutated pre element (opre) and the self-inactivating mutation in the 3' LTR (ΔU3) as indicated (not to scale).

We constructed and validated several designs of LV containing single or multiple combinations of the genes encoding cytokines and antigens required to genetically reprogram monocytes into DC (Figure 1). The LV were derived from a multiply attenuated 3rd generation 4-plasmid split genome packaging system [26]. This vector system remains as a broadly employed HIV-derived lentiviral packaging system for clinical development. *Tat* and the four accessory genes of HIV were deleted from the viral packaging system, which consists of four plasmids used to transfect the 293T packaging cell line [26]. A 400 nucleotide deletion was included in the 3' long terminal repeat (LTR) of the backbone vector (which is copied to the 5' LTR upon reverse transcription), thereby abolishing the 5' LTR promoter activity and reducing risk of vector mobilization with the wild type virus [27]. The original lentivirus envelope protein (gp120) restricts the host range, is unstable, and makes production of LV more complex. Therefore, vectors are usually pseudotyped (*i.e.*, encoated with a heterologous envelope protein) with vesicular stomatitis virus glycoprotein (VSV-G). VSV-G is a highly stable protein that is reported to bind to cell surface phospholipids rather than a specific cellular protein receptor, thereby achieving a wide host range.

**Figure 2:**
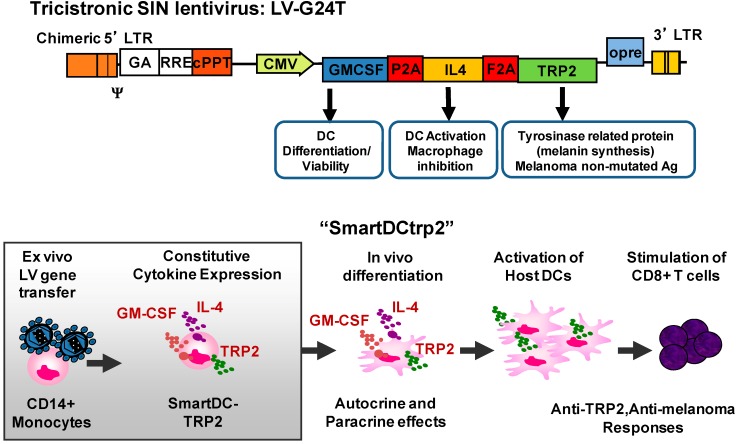
Tricistronic lentiviral vector design validated for production of SmartDCtrp2 *in vivo* in immune competent C57BL6 mice challenged with B16 melanoma and *in vitro* T cell stimulation assays using peripheral blood mononuclear cells from melanoma patients.

Our novel approach was to use these LV designs as an easy and rapid method of genetic reprogramming DC precursors to generate more viable and potent DC [28]. As described in more detail below, we have tested this approach with mouse and human DC precursor cells, with different kinds of promoters driving the expression of cytokines at different levels (early CMV, EF1α, MHCII) exploring integration competent (IC) or integrase-defective vectors (IDLV). Under all these broad different conditions, we could still observe consistent DC self-differentiation initiating 2–3 days after LV transduction. Remarkably, although the levels of cytokines produced by the transduced cell ranged from 0.1 to 50 ng/mL (per 10^6^ cells/mL/24 h), this did not affect the DC reprogramming effect. After one to two weeks after transduction, outgrowth of a very homogeneous DC population could be observed (>90% purity). This strong and consistent effect is likely due to the constitutive production and autocrine use of the cytokines by the monocytes, which renders DC highly viable and phenotypically stable during the life-time of the induced DCs (three to four weeks). Our initial studies testing combinations of lentiviral vectors for transduction of human CD14^+^ DC precursors for co-expression of GM-CSF and IL-4 resulted readily after a few days in a homogeneous culture of “Self-differentiated Myeloid-derived Antigen presenting cells Reactive against Tumors” (SmartDC) [29]. LV expression of a soluble form of FLT3L did not improve induction of monocytes into DCs. Expression of CD40L, on the other hand, fully abrogated DC induction and promoted the formation of large clusters of activated and apoptotic monocytes. Subsequently, using bicistronic vectors co-expressing mouse GM-CSF and IL-4, we confirmed that only these two cytokines were required. Mouse SmartDC were easily produced with non-adherent bone marrow cells after a single overnight exposure to the virus. The day after transduction, the transduced cells could be directly injected subcutaneously into C57BL/6 mice, so that self-differentiation could be completed *in vivo*. SmartDC were highly viable, migrated effectively to lymph nodes, produced LN swelling and resulted in activation of effector cells for several weeks [30]. Co-transduction of DC precursor cells additionally with a vector expressing the mouse tyrosinase related protein 2 (TRP2) or melanoma antigen MART-1 generated potent protective and therapeutic immune responses *in vivo* specific against melanoma [30]. In order to allow co-expression of several cytokines and antigens in a single clinical lentiviral vector, we subsequently developed lentiviral vector designs containing “2A” elements (Figure 1). 2A elements corresponding to 18 amino acid peptide sequences, which serve as co-translational cleavage sites between two protein products. Viruses use these 2A peptides (consensus motif 2A, Asp–Val/Ile–Glu–X–Asn–Pro–Gly; 2B, Pro) to mediate multicistronic expression [31]. Subsequently, we demonstrated that a single tricistronic lentiviral vectors containing sequential 2A elements and expressing TRP2 induced potent murine SmartDCtrp2 (Figure 2), which stimulated anti-melanoma protective and therapeutic effects *in vivo* [32]. Following recommendations from the German regulatory agencies, we tested several different parameters to be taken into consideration the clinical development of human SmartDCtrp2 approach as a novel Advanced Therapy Medicine Product (ATMP) against melanoma. We showed that the transduced cell could be cryopreserved immediately after overnight exposure with LV, without abrogating the *in vitro* or *in vivo* self-differentiation capacity. The quality control criteria of the transduced/cryopreserved cells for batch release are straight-forward, consisting of viability, identity (CD14^+^) and potency (detection of lentiviral copies). As further characterization of the cell product seven days after thawing and *in vitro* culture, we established the immunophenotypic (HLA-DR^+^, CD86^+^, CD14^−^) and cytokine production parameters (GM-CSF, IL-4, TNF-α, MCP-1^+^) [32]. Human SmartDCtrp2 generated with a single tricistronic vector are in development under good manufacturing practices (GMP) for future clinical development [33]. A pilot feasibility batch of the clinical tricistronic vector produced entirely under GMP showed suitable viral titers (5 × 10^8^ infective particles). A SmartDCtrp2 production run applying standard operating procedures (SOPs) under GMP-like conditions was successfully achieved. Two hundred million monocytes isolated by CliniMacs were used for a sequential 28 h cytokine pre-conditioning/lentiviral transduction in bags. After the washing steps, filling, cryopreservation and thawing, 40% of the cells were recovered. After thawing and cell culture for seven days, a homogeneous DC population (CD209^+^, HLA-DR^+^, CD86^+^, CD14^−^) was obtained [34]. Therefore, in just 28 h after the CD14^+^ selection, we expect that more than 50 × 10^6^ cells can be stored as a cryopreserved product. The initial dose of cells for a phase I/IIa clinical trial is planned according to our experience in the murine melanoma model [32]. We expect that a prime/boost dose of 1 × 10^6^ viable thawed cells injected subcutaneously near draining lymph nodes will yield potent immunologic and therapeutic effects.

We also evaluated Wilms’ tumor protein 1 (WT1) as a co-expressed tumor antigen in combination with GM-CSF and IL-4 in tricistronic LV designs. WT1 is a tumor antigen over-expressed in several types of tumors and one of the top antigens currently explored for cancer immunotherapy. However, clinical immunotherapeutic use of WT1 antigenic peptides against different types of neoplasms remains inconclusive. We genetically programmed SmartDC capable of producing and processing endogenous WT1 epitopes using a truncated form of WT1 (lacking the DNA-binding domain). Overnight LV transduction induced monocytes into immunophenotypically stable “SmartDCtWT1”, and the tWT1 protein detectable in DC cytoplasm did not abrogate DC differentiation. SmartDCtWT1 were produced with PBMC obtained from an acute myeloid leukemia patient and also from the matched stem cell donor and used to expand the donor’s CD8^+^ T cells *in vitro*. Expanded CTLs showed antigen-specific reactivity against WT1 and against the primary WT1^+^ leukemia cells. SmartDCtWT1 injected s.c. into Nod.Rag1^−/−^.IL2rγc^−/−^ (NRG) mice were viable *in vivo* for more than three weeks. Migration of human T cells to the immunization site was demonstrated following adoptive transfer of huCTL into mice immunized with SmartDCtWT1. Furthermore, SmartDCtWT1 immunization plus adoptive transfer of T cells reactive against WT1 into mice resulted in growth arrest of a WT1^+^ tumor. Gene array analyses of SmartDCtWT1 demonstrated higher up-regulation of several genes related to innate immunity in comparison to cDC. Thus, SmartDCtWT1 can be produced in a single day of *ex vivo* gene transfer. In the future, it could also be used as a cancer immunotherapeutic product against malignant transformation associated with WT1 over-expression [35].

## 4. Development of Induced Dendritic Cells (iDC) with LV for T Cell Expansion after Stem Cell Transplantation

More than 50,000 hematopoietic stem cell transplants (HSCT) are carried out annually worldwide to treat aggressive diseases that damage or destroy the bone marrow, such as lymphoma, leukemia, multiple myeloma. It can also be used to restore bone marrow that has been damaged by total body radiation and high doses of chemotherapy used for cancer treatment. Doctors consider allogeneic HSCT if the patient has cancer that has come back frequently, such as relapsed leukemia and lymphoma, for which the mortality risk is very high. In past years, peripheral blood stem cell transplantation (PBSCT) has become the most prevailing type of HSCT. The growth factor granulocyte colony stimulating factor (G-CSF) may be used to stimulate the growth of new stem cells from the donor. The blood is removed from the donor’s vein by leukapheresis. The stem cells may be enriched, purified or other cells (T cells, B cells) removed in order to avoid graft-versus-host-disease (GVHD). After infusion into the patient, the transplanted HSC begin to produce new cells in 1 to 3 weeks, but reconstitution of functional T cells may take up to a year and B cell responses up to two years to normalize. Unrelated PBSCT recipients have high risks of developing severe human cytomegalovirus (HCMV) infections and concurrently related complications. This is a major source of severe morbidity, and approaches to accelerate adaptive and long-lasting immunity against HCMV after allo-HCT are warranted [36]. Recently, peptide-pulsed cDC immunotherapy was explored in the acceleration of immune reconstitution against HCMV after allo-HSCT showing in phase I/II clinical trials. Enthusiastic antigen-specific T cell stimulation results were observed, associated with better HCMV control in 25% of the patients and, importantly, no exacerbation of GVHD [37]. Nevertheless, the use of cDC produced from the HSCT donor posed additional production and logistic demands for larger clinical trials.

We therefore initially explored the HCMV tegument phosphoprotein 65 (pp65) as a model viral and immune dominant antigen co-expressed in SmartDCpp65 to stimulate expansion of Ag-reactive donor-derived T cells *in vivo* [38]. We established a human NRG/huPBL model to evaluate the engraftment, expansion and reactivity of human T cells *in vivo*. NRG mice were immunized with SmartDCpp65 and infused with the cognate luciferase-labeled human T cells obtained from the same donor. A dramatic accumulation of T cells was observed at the injection site with subsequent bio-distribution to spleen and other tissues. Immunologic analyses of CD8^+^ T cells recovered from peripheral blood and spleen of these mice confirmed the presence of pp65-reactive human T cells, as analyzed by IFN-γ ELISPOT and tetramer analyses. Conventional DC transduced with LV for pp65 expression did not produce the same effects. Thus, this NRG/huPBL model convincingly demonstrated the requirement of persistent availability of homeostatic cytokines along with antigen-presentation to significantly accelerate human T cell expansion and stimulation in a lymphopenic mouse host. The shortcoming of the NRG/huPBL model was that, 3–4 weeks after infusion of human leukocytes, mice developed lethal GVHD. Therefore, functional antigen-specific immunological analyses were only possible for only up to a month after T cell infusion.

## 5. Integrase-Defective Vectors (IDLV) also Works, and Co-Expression of GM-CSF/IFN-α Works Best for iDC Reprogramming

A well-established target for inhibiting LV integration is mutation of the integrase protein producing integrase-defective lentiviral vectors (IDLV, also known as non-integrating lentiviral vectors, NILV). Although lower levels of transgene expression are detected in comparison to “integrase competent” (IC)LV counterparts, IDLV have been reported to lead to efficient gene expression *in vitro* and *in vivo*, most remarkably in non-dividing cells [39,40]. IDLV are currently in broad development for use as vaccines recombinant, as anti-cancer therapy, to result into site-directed gene insertions or gene disruption strategies, and for cell reprogramming [41]. IDLV can significantly reduce frequency of integration up to a few logs, but there still remains a low level of vector integration, which can be detected by insertion site analysis and high-throughput sequencing. Interestingly, hepatocyte-targeted expression of coagulation factor IX (FIX) using IDLV resulted in the sustained induction of immune tolerance to the transgenic proteins [42]. Despite the reduced transgene expression levels in comparison with their ICLV counterparts, therapeutic FIX expression levels were observed in a mouse model and only rare genomic integrations were observed in hepatocytes [42].

IDLV are particularly attractive in terms of lowering the risk of insertional mutagenesis for genetic reprogramming of human hematopoietic cells, due to the possible risk of leukemogenesis. We used the IDLV platform to compare different cytokine combinations for generation of human iDC: IDLV-SmartDC (co-expressing GM-CSF/IL-4) or IDLV-SmyleDC (co-expressing GM-CSF/IFN-α) [43]. Both types of iDC still produced nanogram/ml levels of transgenic cytokines, showed stable expression of relevant immunophenotypic markers and were autonomously viable *in vivo* for 2–3 weeks after s.c. administration into NRG mice. IDLV-SmyleDC showed higher potency than IDLV-SmartDC. Using pp65 soluble peptides loaded externally, IDLV-SmyleDC did not require additional *in vitro* maturation steps to strongly stimulate autologous T cells, resulting into “licensed antigen presentation” of different pp65 antigenic determinants *in vitro*. When pp65 was lentivirally co-expressed by a second vector in IDLV-smyleDCpp65, were able to expand pp65-reactive adoptive T cells from the same donor in NRG mice. Therefore, IDLV showed long-term expression in non-replicating iDC and replacement of IL-4 by IFN-α resulted in superior activation of DC, bypassing the need of additional maturation steps for optimal antigen presentation [43].

## 6. IDLV-SmyleDCpp65 Produce Remarkable Effects in *de Novo* Adaptive T and B Immune Reconstitution after Stem Cell Transplantation in fully Humanized CD34^+^ Transplanted NRG Mice

**Figure 3:**
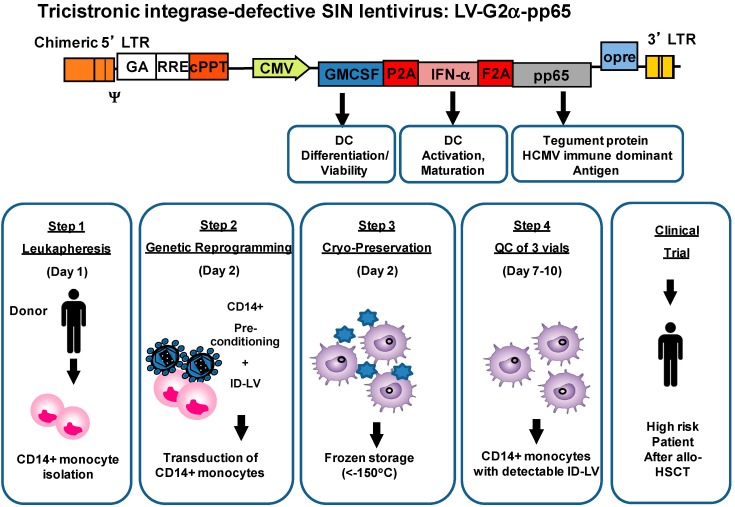
Tricistronic lentiviral vector design validated for production of donor-derived SmyleDCpp65 *in vivo* in humanized mice recapitulating human hematopoietic stem cell transplantation and *in vitro* T cell stimulation assays using peripheral blood mononuclear cells from stem cell donors.

Although the use of iDC could be clinically used to expand adoptively transferred T cells from the allo-HSCT donor in the patient, we were keen to evaluate the feasibility of SmyleDCpp65 to directly prime *de novo* T cells that developed endogenously after allo-HSCT. Similar to the human situation, *de novo* regeneration of immunity is a major problem after xenogeneic HSCT in severely-compromised immune-deficient mice humanized with the human immune system (HIS). CD34^+^ HSC-transplanted HIS-NRG mice do not regenerate their inborn underdeveloped thymus and lymph nodes. Even if hematopoietic cells engraft and proliferate in the bone marrow to high levels (up to 60% human cells in mouse PBL), they show only incomplete maturation of human T and B lymphocytes and adaptive cellular and antibody responses are scarce. We recently demonstrated pre-clinically the immune potency and safety of IDLV-SmyleDCpp65 to generate *de novo* human pp65-reactive T and B effector cells *in vivo* in HIS-NRG mice. We obtained CD14^+^ monocytes and autologous CD34^+^ stem cells isolated from the same peripheral blood of G-CSF mobilized donors, as a clinically relevant stem cell transplantation model. Ten weeks after immunization of humanized mice with SmyleDCpp65, we consistently observed systemic regeneration of peripheral lymph nodes repopulated with terminally differentiated human memory helper, cytotoxic, follicular T helper cells and mature plasma B cells. We demonstrated the adaptive pp65-specific IgG recombination switch and functional human T cell responses against pp65. Despite of the immune responses, some mice showed only minor signs of GVHD [44]. Our results demonstrated in the HIS-NRG model reconstituted with adult CD34^+^ stem cells the significant role of potent and long-lasting SmyleDCpp65 functions after HSCT as a switch for remodeling of peripheral lymph node and lymphatic flow regeneration resulting into activation, mobilization, and finally maturation of lymphocytes towards full immune function. A pilot batch of the IDLV tricistronic vector co-expressing GM-CSF, IFN-α and pp65 was recently produced and purified under standardized GMP-like conditions and the quality control analyses entailed identity, purity, sterility and viral titer (4.2 × 10^9^ infective particles total) [45]. Standardized transduction of monocytes with IDLV for generation of cryopreserved SmyleDCpp65 under GMP-like conditions using bag systems were validated in 3 pre-clinical runs. This served as evidence of feasibility for future clinical development of donor-derived SmyleDCpp65, which can be produced and QC released in a short time (ten days). We are developing the clinical use of SmyleDCpp65 after HSCT to prevent HCMV reactivation in allo-HSCT patients (Figure 3). The specifications for QC and batch release are based on flow cytometry analyses of viable monocytes containing detectable IDLV copies.

## 7. Relevant Functional and Safety Considerations regarding iDC Reprogrammed with IDLV

### 7.1. Viability of cDC versus iDC

As previous discussed, after *ex vivo* generated clinical-grade cDC applied into patients started to be analyzed for viability and migration, a clear outcome was that most of the cells died in the injection sites [4]. Despite of these important observations, additional methods for generating antigen-loaded and mature cDC were still developed, but *in vivo* assays to determine their viability were in most cases still lacking. This is a critical issue in the translation of this type of cell therapy, since DC are post-replicative cells and cannot expand in the host (like for example the case for HSC or T cell therapies). When we proposed the new modality of iDC, an important criteria to continue their development was to evaluate how viable and migratory they were *in vivo*. Mouse iDC were monitored for cDC in immunecompetent C57BL/6 mice by non-invasive optical imaging analyses [30]. We observed that iDC were significantly more viable than cDC, but only if they were allowed to self-differentiate *in vivo*, *i.e.*, the cells were applied subcutaneously shortly after LV transduction. This higher viability correlated with their higher migratory potential and immunogenicity to stimulate T cells against melanoma [30]. Notably, even after iDC reached the draining lymph nodes, they could be detectable by sensitive optical imaging and flow cytometry analyses for three weeks [32]. These results could not be reproduced with cDC. Similarly, human iDC injected subcutaneously into immune deficient mice (but not cDC) showed high viability for up to three weeks [38,43]. In addition, both human and murine iDC could be cryopreserved after LV transduction, thawed and administered, without drastically impacting on their *in vivo* survival [33]. Ultimately, a relevant consequence of iDC high viability in LN of immune competent or humanized was their local effect in the lymphoid tissue, as LN swelling, T cell infiltration and reorganization was frequently observed [30,44]. It is tempting to speculate that iDC applied to humans would recapitulate the findings in humanized mice and induce lymphatic remodeling. In fact, besides feasibility, safety and immunogenicity, this would be a relevant secondary endpoints to assess in Phase I/IIa clinical trials.

### 7.2. Combinations with Other Transgenic Cytokines

Could other combinations of transgenic cytokines (besides GM-CSF plus IL-4 or GM-CSF plus IFN-γ) be effective in iDC reprogramming? As discussed before, GM-CSF is considered a critical factor for DC development under both steady-state and inflammatory conditions [22]. Upon binding to its cognate receptor, GM-CSF activates several intracellular signaling modules, including JAK/STAT, MAPK, PI3K and canonical NF-κB. Cytokines that are complementary to GM-CSF in the downstream STAT or ERK pathways are capable to further stimulate differentiation (IL-4) and activation/maturation of DCs (IFN-γ) [25], and result into different migratory properties. IDLV-mediated expression of GM-CSF plus IFN-γ in human monocytes resulted in more activated iDCs that did not require *in vitro* treatment with additional maturation factors for optimal antigen presentation [43]. Since the multicistronic LV design is combinatorial, we can include additional cytokines that can complement GM-CSF and IFN-γ such as IL-15 (for NK cell stimulation), IL-12 (for optimal Th1 activation), and so on. On the other hand, cytokines that are associated with tolerance (such as IL-10, TGF-B) could be also explored to maintain iDCs in a tolerogenic state. 

### 7.3. Origin of Monocytes

Monocytes should be autologous to the cancer patient or allogeneic obtained from the same G-CSF mobilized donors donating CD34^+^ stem cells for HSCT. They should be highly purified as CD14^+^ cells (>95%). Donors for HSCT are strictly examined for HLA-compatibility, viral infections (HCV, HBV, HIV) and overall health status, making them a safe source of allogeneic monocytes.

### 7.4. Cell Manipulation ex Vivo

After monocytes are isolated by CliniMacs, the full GMP production requires only additional 28 h of *ex vivo* manipulation. Isolated CD14^+^ monocytes are pre-incubated in a bag system with recombinant huGM-CSF/huIL-4 for 8 h, transduced with LV at a low multiplicity of infection (MOI = 5) for 16 h in the presence of protamine sulphate as adjuvant, washed extensively, and cryo-preserved. The *in vitro* transduction of monocytes and the clinical grade of all cell product components provide high levels of safety and conformity. Since the transduction method is short, the emergence of malignant/transformed cells or replication competent lentivirus is unlikely to occur. Up to this date, no replication competent lentivirus (RCL) has been detected in cells transduced *ex vivo* with LV under GMP [46] and there is a current discussion at the FDA regarding RCL testing methods for viral batches. According to the FDA and EMA, cells that are transduced *ex vivo* for less than 4 days do not require RCL testing.

### 7.5. Ability to Proliferate

The transduced monocytes are post-mitotic cells and do not proliferate. *In vitro*, the differentiated DC senesce after 3–4 weeks and we have not observed any malignancy in mice administered s.c. with non-cryopreserved SmyleDCpp65 (maintained in observation for up to 9 months after administration).

### 7.6. Ability to Differentiate into Unwanted Cell Types

Our standardized GMP-like runs demonstrated that the transduced cells differentiate into a homogeneous and pure DC population (>90%). Contamination with other cell types (lymphocytes) was less than 5%.

### 7.7. Immune Toxicity

SmyleDCpp65 stimulated homeostatic and pp65-specific effector memory CD8^+^ and CD4^+^ T cells and humoral responses *in vivo* in a xenograft humanized mouse model recapitulating HSCT. Only mild, grade 1 graft-versus-host disease was observed in 50% of the mice analyzed [44]. More detailed pharm/tox analyses are ongoing.

### 7.8. Mode of Administration

The planned administration route is subcutaneous. The expected outcome is local inflammation and swelling of lymph nodes.

### 7.9. Life Span of Cell

SmyleDCpp65 were highly viable for up to 30 days *in vivo* as shown by analyses performed with optical imaging analyses of SmyleDCpp65 marked with firefly luciferase [43].

### 7.10. Availability of Clinical Data on or Experience with Similar Products

Similar products were not tested in humans, this will be a “first-in-man” evaluation of lentiviral vector-reprogrammed DC in the melanoma immunotherapy or HSCT setting. Conventional DC pulsed with pp65 and p150 peptides showed no increase in acute graft-versus-host disease and a high safety profile in clinical trials [37]. HSCT recipients receiving HSC from haplotype, unrelated or HCMV-donors are at high risk of developing severe HCMV infections. This is a major source of severe morbidity, and approaches to accelerate adaptive and long-lasting immunity against HCMV after allo-HSCT are warranted, and therefore the risk/benefit balance justifies the clinical evaluation.

## 8. Outlook for Future Improvements of iDC and IDLV Immune Therapeutic and Prophylactic Vaccines

The inquisitive reader would probably like to ask whether the *ex vivo* cell modification could be bypassed, *i.e.*, by exploring the direct IDLV administration. This is well possible, although preliminary data generated in our laboratory demonstrated that the direct vaccination with multicistronic IDLV was less effective in the cancer immune therapeutic setting than immunizations with iDCs [47]. In addition, there are still relevant safety concerns regarding the bio-distribution and residual integration of IDLV if it is administered systemically. Non-invasive imaging analyses by our group to track the bio-distribution and persistence if ICLV *in vivo* have demonstrated that VSV-G-pseudotyped vectors expressing firefly luciferase or GFP injected i.v. infected preferentially antigen presenting cells such as DCs and B cells in the spleen, liver and bone marrow [48]. The expression of luciferase persisted for up to 6 months in immune competent mice. Similar studies performed with GFP as marking corroborated the high infectivity of APCs with ICLVs [49]. Systematic analyses of the bio-distribution and residual integration of IDLV vaccine vectors in immune competent or humanized mice are still lacking. Since the effects of direct administration in humans is still unpredictable, it would be important to enhance the safety profile of IDLV by various means, as described below.

### 8.1. Suicide Genes Co-Expressed in the Vector

In feasibility experiments in the mouse system, we have explored a mutated form of the *Herpes Simplex Virus Thymidine Kinase* (*HSV-TK*). When *HSV-TK* was co-expressed in the tricistronic ICLV, murine SmartDC/TK were susceptible to the pro-drug gancyclovir (GCV) and iDC ablation was feasible *in vitro* and *in vivo* [32]. Besides *HSV-TK*, other suicide genes, such as *caspase 9* could be explored as previously described for T cells genetically modified for expression of CAR [18].

### 8.2. Transcriptional Targeting for DC Subset Precursors

A shortened version of the major histocompatibility II (MHCII) promoter was evaluated in multicistronic ICLV co-expressing GM-CSF and IL-4. Since the MHCII promoter is up-regulated during DC differentiation, we evaluated its ability to lead to a higher specificity and feed-back regulatory mechanism leading to “pure” SmartDC populations [32]. Although, as expected, this transcriptional targeting resulted in MHCII/SmartDC with higher purity profile *in vitro*, these cells were less stimulatory *in vivo* than the commonly used CMVpromoter/SmartDC. The mechanistic reasons for this discrepancy are unclear as both types of cells were equally viable and stable *in vitro* and *in vivo*. Other promoters that are selectively active or up-regulated in DCs could be explored, such as Langerin, CD83, CD86 CD80, *etc*.

### 8.3. Pseudotyping IDLV with other Envelopes for DC Targeting

An interesting innovative approach is the use of nano-bodies (Nbs) to target lentivectors to antigen-presenting cells [50]. Nbs are small antibody fragments engineered from heavy-chain-only antibodies found in Camelidae. They were explored as targeting moieties to enable ICLV to infect human myeloid DCs. It would be tempting to use similar approaches to optimize the delivery of multicistronic IDLV to human DC precusors *in vivo*, such that iDC reprogramming would occur *in situ*.

## 9. Conclusions

Our pre-clinical work with iDC in *in vivo* syngeneic and humanized mouse models has shown high viability, bio-distribution, potent immune therapeutic effects and good safety performance. We have not observed signs of carcinogenesis in C57BL/6 or HIS-NRG mice. This is expected, as iDC are not replicating cells, terminally differentiated, with a life span of approximately 3 weeks. The *in vitro* transduction of monocytes and the clinical grade of all vaccine components are likely to provide a high level of safety and conformity. The clinical iDC development will generate know-how for GMP production of IDLV, GMP transduction of monocytes in a bag systems, cryopreservation, administration into patients and immune monitoring. These methodologies can be standardized, optimized and automatized for future phase III clinical trials. This “Advanced Cell Therapy Product” might become a valuable tool for Ag-specific immune regeneration of transplanted and/or immune compromised cancer patients. In addition, these clinical trials and technologies will be, in due time, also adapted for the direct use of multicistronic IDLV as vaccines.
